# Altered Insular Subregional Connectivity Associated With Cognitions for Distinguishing the Spectrum of Pre-clinical Alzheimer's Disease

**DOI:** 10.3389/fnagi.2021.597455

**Published:** 2021-02-10

**Authors:** Siyu Wang, Haiting Sun, Guanjie Hu, Chen Xue, Wenzhang Qi, Jiang Rao, Fuquan Zhang, Xiangrong Zhang, Jiu Chen

**Affiliations:** ^1^Institute of Neuropsychiatry, The Affiliated Brain Hospital of Nanjing Medical University, Fourth Clinical College of Nanjing Medical University, Nanjing, China; ^2^Fourth Clinical College of Nanjing Medical University, Nanjing, China; ^3^Department of Pediatrics, Xijing Hospital, The Fourth Military Medical University, Xi'an, China; ^4^Institute of Brain Functional Imaging, Nanjing Medical University, Nanjing, China; ^5^Department of Radiology, The Affiliated Brain Hospital of Nanjing Medical University, Nanjing, China; ^6^Department of Rehabilitation, The Affiliated Brain Hospital of Nanjing Medical University, Nanjing, China; ^7^Department of Psychiatry, The Affiliated Brain Hospital of Nanjing Medical University, Nanjing, China; ^8^Department of Geriatric Psychiatry, The Affiliated Brain Hospital of Nanjing Medical University, Nanjing, China

**Keywords:** amnestic mild cognitive impairment, subjective cognitive decline, fMRI, insular subnetwork, functional connectivity, episodic memory, executive function

## Abstract

**Background:** Subjective cognitive decline (SCD) and amnestic mild cognitive impairment (aMCI) are regarded as part of the pre-clinical Alzheimer's disease (AD) spectrum. The insular subregional networks are thought to have diverse intrinsic connectivity patterns that are involved in cognitive and emotional processing. We set out to investigate convergent and divergent altered connectivity patterns of the insular subregions across the spectrum of pre-clinical AD and evaluated how well these patterns can differentiate the pre-clinical AD spectrum.

**Method:** Functional connectivity (FC) analyses in insular subnetworks were carried out among 38 patients with SCD, 56 patients with aMCI, and 55 normal controls (CNs). Logistic regression analyses were used to construct models for aMCI and CN, as well as SCD and CN classification. Finally, we conducted correlation analyses to measure the relationship between FCs of altered insular subnetworks and cognition.

**Results:** Patients with SCD presented with reduced FC in the bilateral cerebellum posterior lobe and increased FC in the medial frontal gyrus and the middle temporal gyrus. On the other hand, patients with aMCI largely presented with decreased FC in the bilateral inferior parietal lobule, the cerebellum posterior lobe, and the anterior cingulate cortex, as well as increased FC in the medial and inferior frontal gyrus, and the middle and superior temporal gyrus. Logistic regression analyses indicated that a model composed of FCs among altered insular subnetworks in patients with SCD was able to appropriately classify 83.9% of patients with SCD and CN, with an area under the receiver operating characteristic (ROC) curve (AUC) of 0.876, 81.6% sensitivity, and 81.8% specificity. A model consisting of altered insular subnetwork FCs in patients with aMCI was able to appropriately classify 86.5% of the patients with aMCI and CNs, with an AUC of 0.887, 80.4% sensitivity, and 83.6% specificity. Furthermore, some of the FCs among altered insular subnetworks were significantly correlated with episodic memory and executive function.

**Conclusions:** Patients with SCD and aMCI are likely to share similar convergent and divergent altered intrinsic FC patterns of insular subnetworks as the pre-clinical AD spectrum, and presented with abnormalities among subnetworks. Based on these abnormalities, individuals can be correctly differentiated in the pre-clinical AD spectrum. These results suggest that alterations in insular subnetworks can be utilized as a potential biomarker to aid in conducting a clinical diagnosis of the spectrum of pre-clinical AD.

## Introduction

Subjective cognitive decline (SCD) and amnestic mild cognitive impairment (aMCI), which are part of the clinical continuum of dementia progression and the spectrum of pre-clinical Alzheimer's disease (AD), are well-established risk factors for the development of AD (van der Flier et al., 2004; Stewart et al., 2008; Striepens et al., 2010; Scheef et al., 2012; Jessen et al., 2014b, 2020; Buckley et al., 2016; Xue et al., 2019). Many studies have suggested that individuals with SCD go on to develop aMCI, and then AD (Jessen et al., 2014b; Berger-Sieczkowski et al., 2019). Therefore, our in-depth understanding of neuroimaging-based continual pathology across the spectrum of pre-clinical AD can help assist in the development of a new method in pre-clinical AD diagnosis and treatment.

Neuroimaging can help reveal the pathological mechanisms behind AD progression. The insula and its network connectivity are anatomically connected to the limbic and frontal-parietal-temporal lobes and are functionally involved in a higher-order cognitive and emotional processing (Allen et al., 2008; Naqvi and Bechara, 2010), which has a significant role in AD progression (Fan et al., 2008; Guo et al., 2012). Numerous studies have reported the presence of insular gray matter atrophy in AD (Guo et al., 2012), as well as altered insular activities and networks in aMCI (Xie et al., 2012; Lin et al., 2017). In particular, studies have demonstrated that insula atrophy can help correctly distinguish patients with AD from normal controls (CNs; Fan et al., 2008; Guo et al., 2012). Therefore, it is reasonable to hypothesize that the insula is an extremely vulnerable area, as well as a critical hub, in delaying the progression of the pre-clinical spectrum of AD, including SCD and aMCI.

Recently, several studies have consistently suggested that the insula demonstrates heterogeneity and may be divided into the ventral anterior insula (vAI), the dorsal anterior insula (dAI), and the posterior insula (PI) (Deen et al., 2011; Peng et al., 2018; Lu et al., 2020). These insula subregional networks have different roles in information processing (Taylor et al., 2009) and therefore, have distinct intrinsic connectivity patterns (Deen et al., 2011; Peng et al., 2018; Lu et al., 2020). Studies have demonstrated that patients with aMCI and AD present with distinct disruption in the connectivity of insular subregions (Xie et al., 2012; Liu et al., 2018; Lu et al., 2020), which is associated with episodic memory deficits (Xie et al., 2012). Increasing evidence indicates that insular subregional networks can reveal neuroimaging-based continuum pathology across the spectrum of pre-clinical AD in a sensitive and specific manner. However, there is still a lack of knowledge with regards to altered network connectivity patterns of the insular subregions across the spectrum of pre-clinical AD (SCD and aMCI), as well as its relationship with cognition. In particular, it is unclear whether altered connectivity patterns of the insula subregions can help distinguish the spectrum of pre-clinical AD.

We conducted this study to identify altered intrinsic connectivity patterns of the insular subregions across the spectrum of pre-clinical AD. After identification of intrinsic connectivity pathology, we further evaluated how well this can be used to distinguish the spectrum of pre-clinical AD. Finally, we hypothesized that patients with SCD and aMCI present with different altered intrinsic connectivity patterns of the insular subregions. Additionally, we further hypothesized that integration of altered intrinsic functional connectivity (FC) can accurately distinguish individuals in the spectrum of pre-clinical AD.

## Materials and Methods

### Participants

The data used in this study were acquired from our in-home database, the Nanjing Brain Hospital-Alzheimer's Disease Spectrum Neuroimaging Project (NBH-ADsnp) (Nanjing, China), which is continuously updated. The relevant information obtained from the NBH-ADsnp is summarized in Supplementary Methods S.1. This study was granted approval by the responsible Human Participants Ethics Committee of the Affiliated Brain Hospital of Nanjing Medical University (Nos. 2018-KY010-01 and 2020-KY010-02), located in Nanjing, China. Signed informed consent was obtained from each participant.

Initially, 460 individuals, who were of Han Chinese origin and right-handed, were enrolled in the NBH-ADsnp database from the memory clinic of hospitals and local communities through the use of advertising and broadcasting. All patients with SCD (*n* = 116) and CNs (*n* = 190) were recruited from local communities, while aMCI subjects (*n* = 154) were enrolled from both memory clinics and local communities. According to our criteria, a total of 169 elderly individuals participated in this study. Among them, two CNs, two patients with SCD, and 16 patients with aMCI were excluded due to excessive head movement during MRI scan (defined as cumulative translation or rotation of >3.0 mm or 3.0° and individual time points with mean frame-wise displacement (FD) of >0.5 mm and scans with >50% volumes removed; Brady et al., 2019), and incomplete or missing MRI data. The remaining 55 CNs, 38 patients with SCD, and 56 patients with aMCI were enrolled in the final study. The inclusion and exclusion criteria were outlined in accordance with previously published studies (Jessen et al., 2014a, 2020; Chen et al., 2015a, 2016a, 2019a; Dubois et al., 2016; Xue et al., 2019). The diagnosis was performed independently by three neuropsychiatric physicians (Dr. Jiang Rao, Fuquan Zhang, and Xiangrong Zhang) who had 12–15 years of experience. Any disagreement between the three neuropsychiatric physicians was solved by reaching a consensus through group discussions.

The inclusion criteria of SCD subjects included the published SCD research criteria proposed by the Subjective Cognitive Decline Initiative (SCD-I; Jessen et al., 2014a). The detailed inclusion criteria were described in our previously published study (Xue et al., 2019), and is as follows: (a) self-reported persistent memory decline, confirmed by the caretaker or a family member; (b) a Subjective Cognitive Decline Questionnaire (SCD-Q) score > 5 (Hao et al., 2017; Yan et al., 2018; Cedres et al., 2019); (c) performance within a normal range on the Mini-Mental State Examination (MMSE) and the Montreal Cognitive Assessment (MoCA) (adjusted for age and education); (d) Clinical Dementia Rating (CDR) = 0; (e) subjects must be between the ages of 50 and 80; and (f) subjects must have Hamilton Depression Scale (HAMD) scores of < 7.

The inclusion criteria of aMCI subjects included the diagnostic criteria that were previously defined by Petersen et al. (1999) and the revised consensus standards reported by Winblad et al. (2004). The detailed inclusion criteria have been previously described in our published studies (Chen et al., 2016b, 2019a; Xue et al., 2019) and includes: (a) complaint of loss of memory, preferably corroborated by an informant or the subject for more than 3 months; (b) objective memory impairment (adjusted for age and educational level); (c) normal general cognitive function with MMSE score ≥ 24; (d) no or minimal impairment in daily life activities; (e) CDR = 0.5; (f) subjects must be between the ages of 50 and 80; (g) absence of dementia symptoms not sufficient to meet the criteria of the National Institute of Neurological and Communicative Disorders and Stroke or the AD and Related Disorders Association criteria for AD; and (h) subjects must have HAMD scores of < 7.

The inclusion criteria of CN were as follows: (a) no complaints of loss of memory; (b) normal cognitive performance (matched for age and education); (c) CDR = 0; (d) MMSE ≥ 26; (e) subjects must be between the ages of 50 and 80 (Chen et al., 2019b; Xue et al., 2019); and (f) subjects must have a HAMD score of < 7.

Detailed exclusion criteria for all subjects were described in our previously published studies (Chen et al., 2016b, 2019a; Xue et al., 2019), and included (a) past history of stroke (modified Hachinski Ischemic Scale score > 4), alcoholism, head injury, brain tumors, Parkinson's disease, epilepsy, encephalitis, major depression (excluded by HAMD), or other neurological or psychiatric diseases (excluded by clinical assessment and case history); (b) diagnosis of a major medical illness (i.e., cancer, anemia, thyroid dysfunction, syphilis, or HIV); (c) severe loss of vision or hearing; (d) inability to complete neuropsychological tests or a contraindication for MRI, and (e) T2-weighted MRI displaying major changes in white matter (WM), infarction, or additional lesions, as determined by the scan analysis done by two experienced radiologists. None of the subjects used any medications.

All participants underwent a standardized clinical evaluation protocol, including sequencing, demographic inventory, medical history, neurological and mental status examination, and MRI scan. The neuropsychologic tests were conducted by two different neurologists (Dr. Jiang Rao and Dr. Chen Xue) who had 3–5 years of experience. Specific demographics and neuropsychological characteristics of each group are provided in Table 1.

**Table 1:** Demographics, clinical measures, and head rotation parameters of aMCI, SCD, and CN subjects.

**Characteristics**	**CN**	**SCD**	**aMCI**	***F-*values (**χ^2^**)**	***p-*values**
	***n* = 55**	***n* = 38**	***n* = 56**		
Age (years)	62.91 (5.94)	65.84 (7.73)	64.30 (7.70)	1.927	0.149
Gender (male/female)	23/32	8/30	15/41	5.243	0.074
Education level (years)	12.51 (2.51)	12.22 (2.72)	11.15 (2.87)^b^	3.813	0.024^*^
MMSE	28.58 (1.43)	28.32 (2.63)	27.29 (1.59)^b^^,^^c^	11.426	0.000^*^
MoCA	25.05 (2.42)	24.92 (1.79)	22.73 (2.88)^b^^,^^c^	14.787	0.000^*^
MDRS	141.46 (2.33)	140.37 (3.05)	136.45 (6.67)^b^^,^^c^	17.874	0.000^*^
SCD-Q	3.55 (1.50)	6.51 (0.90)^a^	5.06 (1.79)^b^^,^^c^	44.589	0.000^*^
HAMD	1.82 (2.26)	3.92 (3.17)^a^	3.80 (3.64)^b^	7.614	0.001^*^
**Composite Z scores of each cognitive domain**					
Episodic memory	0.27 (0.53)	0.34 (0.59)	−0.49 (0.61)^b^^,^^c^	33.177	0.000^*^
Information processing speed	0.27 (0.67)	0.18 (0.71)	−0.38 (0.74)^b^^,^^c^	13.325	0.000^*^
Executive function	0.27 (0.48)	0.30 (0.57)	−0.46 (0.62)^b^^,^^c^	31.026	0.000^*^
Visuospatial function	0.17 (0.66)	0.26 (0.50)	−0.34 (0.96)^b^^,^^c^	9.400	0.000^*^
**Head rotation parameters**					
FD_VanDijk	0.05 (0.03)	0.04 (0.03)	0.04 (0.03)	0.880	0.417
FD_Power	0.18 (0.08)	0.16 (0.09)	0.16 (0.09)	1.046	0.354
FD_Jenkinson	0.09 (0.04)	0.09 (0.05)	0.08 (0.05)	0.667	0.515

Data are presented as mean (standard deviation, SD). CN, normal control; SCD, subjective cognitive decline; aMCI, amnestic mild cognitive impairment; MMSE, Mini-Mental State Examination; MoCA, Montreal Cognitive Assessment; MDRS, Mattis Dementia Rating Scale; HAMD, Hamilton Depression Scale; CDR, Clinical Dementia Rating; SCD-Q, Subjective Cognitive Decline-Questionnaire; FD, frame-wise displacement.

* Significant differences were found among CN, SCD, and aMCI subjects. Most p-values were obtained using ANOVA, except for gender (chi-square test). Comparisons of each paired group were conducted to further reveal the source of ANOVA difference (

a : SCD vs. CN;

b : aMCI vs. CN;

c : aMCI vs. SCD

*) Bonferroni correction was applied for multiple group comparisons. MMSE, MoCA, and MDRS were displayed as raw scores. This study utilized the composite Z scores to determine the level of each cognitive domain. Supplementary Table 2 displays detailed raw scores and the corresponding Z scores of neuropsychological assessments*.

### Neuropsychological Assessment

Neuropsychological assessments were similar to those used in our previous studies (Chen et al., 2015a, 2016a, 2019a,b; Xue et al., 2019). These assessments were utilized for evaluating the general cognitive function, episodic memory, speed of information processing, executive function, and visuospatial function. Details regarding each of the assessments are listed in Supplementary Methods.

### MRI Data Acquisition

The detailed parameters of MRI acquisition of the NBH-ADsnp are summarized in Supplementary Methods. Imaging analysts were blinded to the status of diagnosis.

### Functional MRI Data Pre-processing

We utilized MATLAB2015b and DPABI software (Yan et al., 2016) to preprocess all functional MRI (fMRI) data. The image processing procedure was conducted as previously described (Yan et al., 2013) and is summarized in Supplementary Methods. In brief, the first 10 time-points were removed, and the data were adjusted for time and motion effects with several nuisance variables [Friston 24-parameter model, with WM, cerebrospinal fluid (CSF), global signals and the linear trend removed; Ashburner and Friston, 2009]. Then, the functional images were spatially normalized, smoothed, and temporal band-pass filtered. The voxels within a group gray matter (GM) mask created by DARTEL were used for further analyses.

### Structural MRI Data Pre-processing

Structural MRI analysis was carried out during fMRI data preprocessing using the DPABI image processing software. First, we manually reoriented and shifted the structural images to define the anterior commissure as the origin. Next, we used the DARTEL technique for normalization and segmentation, which divided the structural images into GM, WM, and CSF (Almairac et al., 2018), and created a group-specific GM (functional connection analysis was restricted to voxels within the GM mask) and WM template. Then, the native and DARTEL versions were provided for GM, WM, and CSF tissues, and native versions were utilized to compute the global intracranial volumes (ITV).

### Quality Assurance

#### The Effect of Brain Atrophy

Given that significant GM atrophies that are present within patients with SCD (Scheef et al., 2012) and aMCI (Trzepacz et al., 2013; Chen et al., 2015a,b, 2020), the anatomical differences between each group may affect the FCs of insular subregions. In order to investigate this issue, we computed ITV based on native GM, WM, and CSF in CN, SCD, and aMCI groups through the use of in-home MATLAB codes. We explored the differences among each group on the FC of insular subnetworks using ITV as an additional covariate in the general linear model (GLM) analysis.

#### Head Motion Effect

We used three different approaches to control for head motion effect, both at an individual and group level. First, we excluded CNs and patients with SCD and aMCI that had excessive head motion (defined as cumulative translation or rotation >3.0 mm or 3.0°). Next, a Friston 24-parameter model was applied for regressing out the effects of head motion from the realigned data (Friston et al., 1996). Then, a “scrubbing” procedure was performed to scrub frames (volumes) that had an excessively high whole-brain root mean square (RMS) signal change over time in the preprocessed fMRI data for each participant (Sheline et al., 2010; Power et al., 2012; Van Dijk et al., 2012). Furthermore, any volume with an FD that was > 0.2 mm as nuisance covariates was regressed out, and scans with 50% of volumes were removed and discarded, as previously described in a study (Brady et al., 2019). Overall, two CNs, two patients with SCD, and 16 patients with aMCI were excluded on the account of excessive head movement. There were no significant changes with regards to between-group differences found in quality assurance parameters of head motion in qualified subjects (Table 1).

#### Strict Multiple Comparison Correction Strategy

In order to ensure reproducibility, test-retest reliability, and replicability using the fMRI metrics, we conducted a strict multiple comparison correction (Chen et al., 2018). That is, statistical maps were set with a threshold applying the permutation test with Threshold-Free Cluster Enhancement (TFCE; Smith and Nichols, 2009) and the family-wise error (FWE), as was implemented in DPABI (Yan et al., 2016).

##### Definition of Insular Subregions

For this study, we created six 6-mm radius spherical seeds. Our definition of insular subregions was in accordance with recent studies (Deen et al., 2011; Peng et al., 2018; Lu et al., 2020). Essentially, the insula was separated into six subregions, which included the left and right bilateral ventral anterior insula (L-vAI and R-vAI), the left and right bilateral dorsal anterior insula (L-dAI and R-dAI), and the left and right bilateral posterior insula (L-PI and R-PI) (Peng et al., 2018; Lu et al., 2020). Supplementary Table 1 details the Montreal Neurological Institute (MNI) seed coordinates.

### Functional Connectivity Analyses

For each participant, we extracted the average time courses for all voxels within each insula subregional seed and used that as the reference time course. Next, we conducted voxel-wise cross-correlation analysis between the averaged time courses of all voxels within the insular subregional seed, as well as each voxel in the remainder of the entire brain within the group-specific GM mask. Finally, we increased the normality of correlation coefficients, and a Fisher's z-transformation analysis was performed.

### Statistical Analyses

#### Demographics and Neuropsychological Data

ANOVA and chi-square tests were conducted for a comparison of differences of the demographic data, clinical measures, and head rotation parameters among the SCD, aMCI, and CN subjects. Notably, in order to improve the statistical power of the study, we applied a re-sampling method of stationary bootstrap (10,000 bootstrap samplings) to obtain significance.

We also set out to improve statistical power by diminishing random variability, as was done in a previous study (Chen et al., 2016b). This was done by compositing neuropsychological assessments into four cognitive domains (episodic memory, information processing speed, executive function, and visuospatial function), and the raw scores were then transformed into four composite Z scores. The details of raw and composite scores for the neuropsychological tests are outlined in Supplementary Table 2.

#### Identification of Altered Connectivity Patterns of the Insula Subregions

Using GLM analysis, we evaluated the differences of the FCs of insula subregions among subjects with aMCI, SCD, and CN (1,000 permutations, TFCE-corrected; *p* < 0.001 and cluster size > 227 mm^3^). Then, we constructed a mask based on the different brain regions. Comparisons between any two groups (i.e., CN vs. SCD, CN vs. aMCI) were performed under this mask after adjusting for age, sex, education, ITV, and mean FD (1,000 permutations, TFCE-FWE-corrected *p* < 0.05, and cluster size > 227 mm^3^).

#### Classification Based on Altered Connectivity Patterns of Insular Subregions

In order to further identify the FC patterns of insular subregions, closely related to patients with SCD and aMCI, we applied an approach based on binary logistic regression analysis using alterations in the identified ROIs as biomarkers to evaluate how well these can help distinguish patients with SCD or aMCI from CN subjects. The receiver operating characteristic (ROC) curve was applied to determine the classification power of this model, as well as to assess its accuracy, sensitivity, and specificity.

#### Relationship Between Altered Connectivity Patterns of the Insula Subregions and Cognition

In order to empirically verify the behavioral significance of altered FC within insular subnetworks in patients with SCD and aMCI, a Pearson's correlation analysis was performed to calculate the relationship between abnormal FC patterns of insula subregions, cognition, and clinical measure (HAMD scores) after adjusting for age, sex, and education. Furthermore, we assessed the significance of these correlation analyses through the use of a leave-one-out cross-validation procedure in 10,000 Monte-Carlo simulation tests to measure how often these results would be expected by chance in a study of multiple comparisons and a re-sampling method of stationary bootstrap (10,000 bootstrap samplings) in order to obtain significance.

## Results

### Demographic and Neuropsychological Characteristics

We found no significant differences with regard to age and gender across each of the three groups (*p* > 0.05; Table 1). In comparison with CNs, patients with aMCI had significantly decreased education level (*p* < 0.05), while patients with SCD were not significantly different. In comparison to CNs, patients with SCD only showed significantly increased SCD-Q and HAMD scores, while patients with aMCI exhibited significantly reduced MMSE, MoCA, and MDRS scores, higher SCD-Q and HAMD scores, and a significant decline in executive function, episodic memory, information processing speed, and visuospatial cognition (*p* < 0.05).

### Functional Connectivity of Insular Subnetworks in Patients With SCD and aMCI

**Figure 1:**
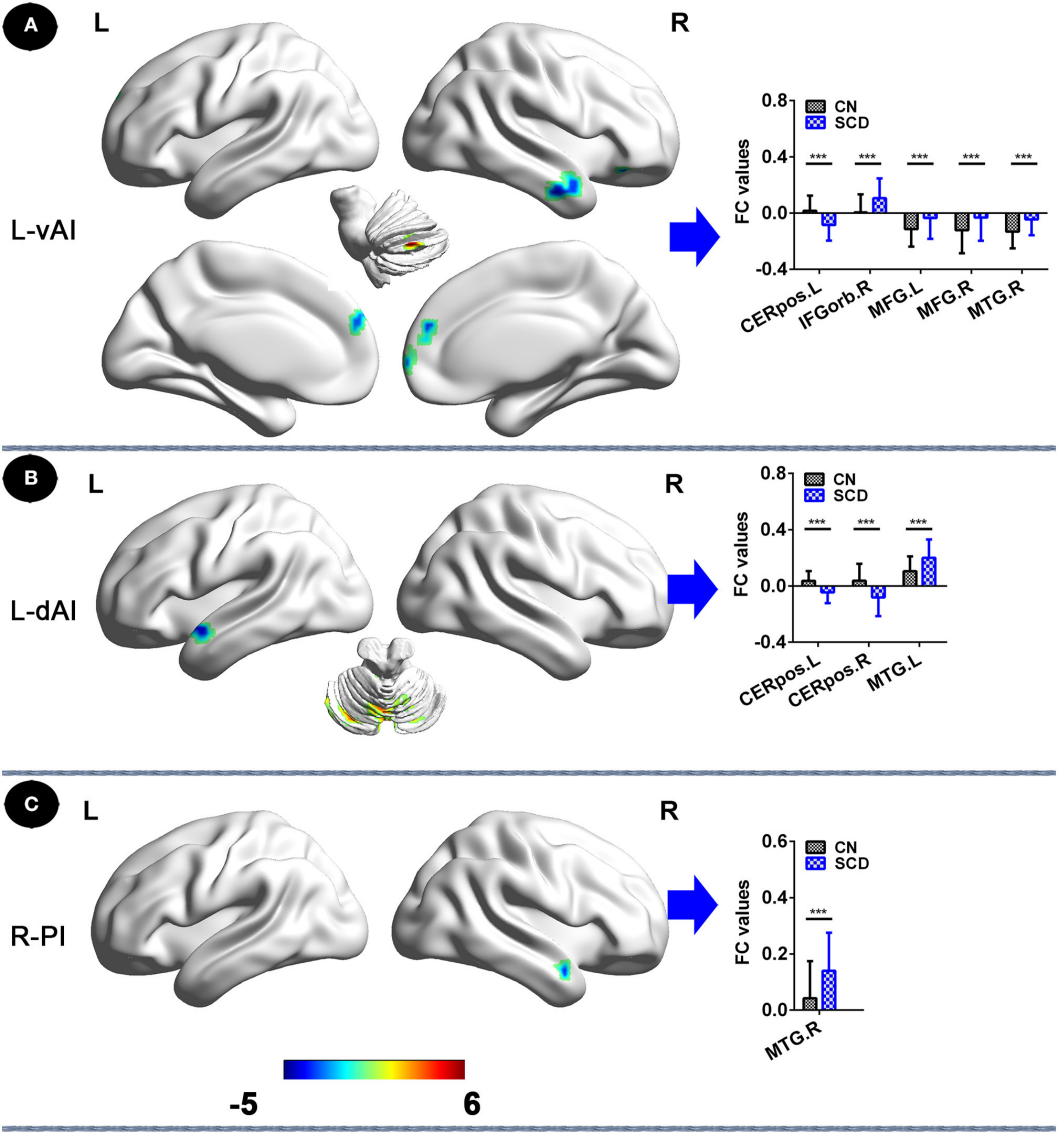
Insular subnetwork functional connectivity in patients with SCD compared to CNs. **(A)** The different brain regions of the FC of the left vAI subregion between CN and SCD. A bar chart indicating the quantitative comparison of functional connectivity between these regions. **(B)** FC of left dAI subregion between CN and SCD. A bar chart indicating the quantitative comparison of FC between these regions. **(C)** FC of the right PI subregion between CN and SCD. A bar chart indicating the quantitative comparison of FC between these regions. *P_TFCE−FWE_ < 0.05. Note: only subnetworks that show significant differences in between-group comparisons are shown here. All results are displayed after adjusting for age, sex, education, ITV, and FD. A threshold of *p* < 0.05 was applied, and TFCE-FWE correction with cluster size >270 mm^3^. FC, functional connectivity; CN, normal control; SCD, subjective cognitive decline; L-vAI, left ventral anterior insula; L-dAI, left dorsal anterior insula; R-PI, right posterior insula; TFCE, threshold-free cluster enhancement; FEW, family-wise error; ITV, intracranial volume; FD, frame-wise displacement; CERpos.L, left cerebellum posterior lobe; CERpos.R, right cerebellum posterior lobe; MTG.R, right middle temporal gyrus; IFGorb.R, right inferior frontal gyrus, orbital part; MFG.R, right medial frontal gyrus; MFG.L, left medial frontal gyrus; MTG.L, left middle temporal gyrus.

In the left vAI subnetwork, in comparison to CNs, patients with SCD displayed reduced FC in the left cerebellum posterior lobe and increased FC in the bilateral medial frontal gyrus (MFG), the right middle temporal gyrus (MTG), and the right inferior frontal gyrus (orbital part) (IFGorb) (*P*_TFCE−FWE_ < 0.05, cluster size > 227 mm^3^) (Figure 1A, Table 2). Additionally, in the left dAI subnetwork, compared to CNs, patients with SCD showed decreased FC in the bilateral cerebellum posterior lobe and the left cerebellum anterior lobe, with increased FC in the left MTG (*P*_TFCE−FWE_ < 0.05, cluster size > 227 mm^3^) (Figure 1B, Table 2). Furthermore, in the right PI subnetwork, patients with SCD exhibited increased FC in right MTG in comparison with CNs (*P*_TFCE−FWE_ < 0.05, cluster size > 227 mm^3^) (Figure 1C, Table 2).

**Table 2:** Comparisons of functional connectivity of insular subregions among aMCI, SCD, and CN subjects.

**Brain regions**	**L/R**	**BA**	**MNI**			***F*/*T*-values**	**Cluster size (mm^**3**^)**
			**x**	**y**	**z**		
**L-vAI FUNCTIONAL CONNECTIVITY**							
**CN vs. SCD**							
Cerebellum posterior lobe	L		−51	−63	−33	5.7672	864
Middle temporal gyrus	R	21	60	−9	−27	−3.8771	2,241
Inferior frontal gyrus, orbital part	R	11	39	33	−15	−4.0899	324
Medial frontal gyrus	R	10	6	63	3	−3.3068	432
Medial frontal gyrus	L	9	6	51	24	−3.3713	1,026
**CN vs. aMCI**							
Medial frontal gyrus	L/R	11	−9	54	−15	−4.0347	14,121
Middle temporal gyrus	R	11	63	−63	3	−3.6545	297
Superior temporal gyrus	R	39	51	−57	21	−3.7118	297
Inferior parietal lobule	R	40	33	−54	42	4.7854	864
Inferior parietal lobule	L	40	−42	−51	51	3.8108	1,728
**SCD vs. aMCI**							
None							
**L-dAI FUNCTIONAL CONNECTIVITY**							
**CN vs. SCD**							
Cerebellum posterior lobe	R		36	−60	−57	4.1111	486
Cerebellum posterior lobe/Cerebellum anterior lobe	L		3	−60	−18	4.5395	15,147
Middle temporal gyrus	L	38	−51	3	−12	−4.5773	405
**CN vs. aMCI**							
Cerebellum posterior lobe	L		−33	−87	−27	3.4273	2,025
Cerebellum posterior lobe/Cerebellum anterior lobe	L		−6	−69	−21	3.5586	2,052
Anterior cingulate cortex	L/R	24	0	15	24	4.1218	567
**SCD vs. aMCI**							
None							
**R-PI FUNCTIONAL CONNECTIVITY**							
**CN vs. SCD**							
Middle temporal gyrus	R	21	48	0	−24	−4.0283	297
**CN vs. aMCI**							
Inferior frontal gyrus, orbital part	R	47	42	21	−3	−3.7377	2,160
Inferior frontal gyrus, triangular part	R	46	51	33	15	−3.2717	540
**SCD vs. aMCI**							
None							

*CN, normal control; SCD, subjective cognitive decline; aMCI, amnestic mild cognitive impairment; TFCE, threshold-free cluster enhancement; FEW, family-wise error; ITV, intracranial volume; FD, frame-wise displacement; L-vAI, left ventral anterior insula; L-dAI, left dorsal anterior insula; R-PI, right posterior insula; MNI, Montreal neurological institute; L, left hemisphere; R, right hemisphere. To note, only the subnetworks that have significant between-group differences are shown here. All results are displayed after adjusting for age, sex, education, ITV, and FD at a threshold of p < 0.05, after applying TFCE-FWE correction with cluster size > 270 mm^3^*.

**Figure 2:**
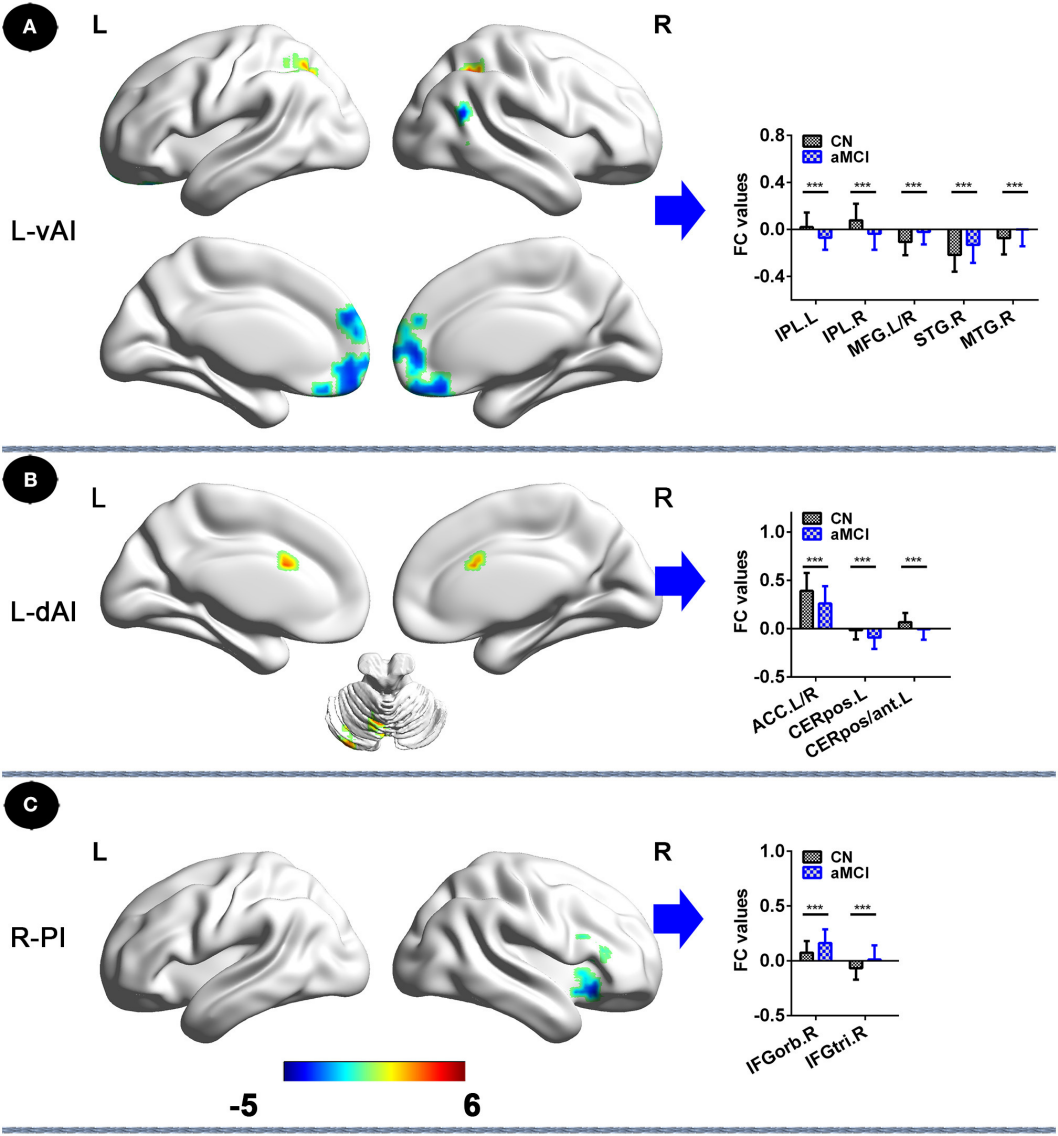
Insular subnetwork functional connectivity in patients with aMCI compared to CNs. **(A)** FC of left vAI subregion between CN and aMCI patients. A bar chart indicating the quantitative comparison of FC between these regions. **(B)** Functional connectivity of the left dAI subregion between CN and aMCI patients. A bar chart indicating the quantitative comparison of FC between these regions. **(C)** FC of right PI subregion between CN and aMCI patients. A bar chart indicating the quantitative comparison of FC between these regions. *P_TFCE−few_ < 0.05. Note: only the subnetworks that show between-group differences are shown here. All results are displayed after adjusting for age, sex, education, ITV, and FD. A threshold of *p* < 0.05 was applied, with a TFCE-FWE correction with cluster size > 270 mm^3^. FC, functional connectivity; CN, normal controls; aMCI, amnestic mild cognitive impairment; L-vAI, left ventral anterior insula; L-dAI, left dorsal anterior insula; R-PI, right posterior insula; TFCE, threshold-free cluster enhancement; FEW, family-wise error; ITV, intracranial volume; FD, frame-wise displacement; IPL.L, left inferior parietal lobule; IPL.R, right inferior parietal lobule; STG.R, right superior temporal gyrus; MTG.R, right middle temporal gyrus; MFG. L/R, left/right medial frontal gyrus; ACC.L/R, anterior cingulate cortex; CERpos.L, left cerebellum posterior lobe; CERpos/ant.L, left cerebellum posterior lobe/cerebellum anterior lobe; IFGorb.R, right inferior frontal gyrus, orbital part; IFGtri.R, right inferior frontal gyrus, triangular part.

In the left vAI subnetwork, compared to CNs, patients with aMCI exhibited decreased FC in bilateral inferior parietal lobule (IPL) and increased FC in bilateral MFG, and right, middle, and superior temporal gyrus (STG; *P*_TFCE−FWE_ < 0.05, cluster size > 227 mm^3^) (Figure 2A, Table 2). In addition, in the left dAI subnetwork, compared with CNs, patients with aMCI showed decreased FC in the bilateral cerebellum posterior lobe, the left cerebellum anterior lobe, and the bilateral anterior cingulate cortex (ACC) (*P*_TFCE−FWE_ < 0.05, cluster size > 227 mm^3^) (Figure 2B, Table 2). Finally, in the right PI subnetwork, in comparison with CNs, patients with aMCI displayed increased FC in right IFGrob and IFGtri (triangular part) (*P*_TFCE−FWE_ < 0.05, cluster size > 227 mm^3^) (Figure 2C, Table 2). All results were adjusted for age, sex, education, ITV, and FD.

However, it is important to note that other insular subnetworks did not show significant differences among CN, SCD, and aMCI subjects.

### Classification of SCD, aMCI, and CN Using the Binary Logistic Regression

**Figure 3:**
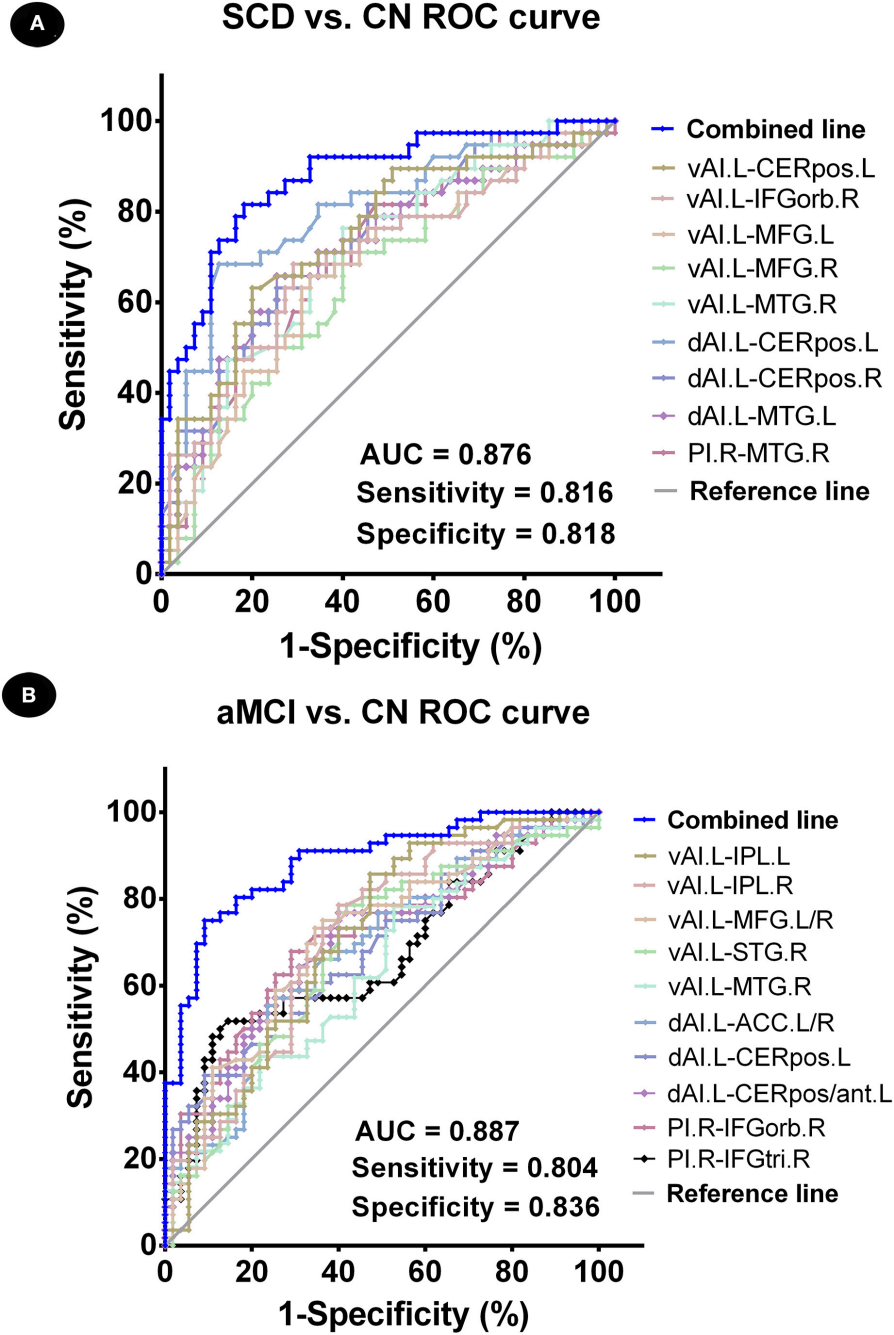
Classification of individuals as SCD vs. CN and aMCI vs. CN using an MRI-based classifier. **(A)** ROC curve showcasing the classification power using an MRI-based “classifier” of SCD from CN. **(B)** ROC curve presetting the classification power using MRI-based “classifier” of aMCI from CN. Note: the values of AUC, sensitivity, and specificity are displayed in the lower right region of the figure. The combined line reflects the combination of indices of abnormal insular subnetwork functional connectivity. CN, normal control; SCD, subjective cognitive decline; aMCI, amnestic mild cognitive impairment; AUC, area under the ROC curve; ROC, receiver operating characteristic; vAI.L, left ventral anterior insula; dAI.L, left dorsal anterior insula; PI.R, right posterior insula; CERpos.L, left cerebellum posterior lobe; CERpos.R, right cerebellum posterior lobe; MTG.R, right middle temporal gyrus; IFGorb.R, right inferior frontal gyrus, orbital part; MFG.R, right medial frontal gyrus; MFG.L, left medial frontal gyrus; MTG.L, left middle temporal gyrus; IPL.L, left inferior parietal lobule; IPL.R, right inferior parietal lobule; STG.R, right superior temporal gyrus; ACC.L/R, anterior cingulate cortex; CERpos/ant.L, left cerebellum posterior lobe/cerebellum anterior lobe; IFGtri.R, right inferior frontal gyrus, triangular part.

Binary logistic regression analyses revealed that the best-fitting model that combined altered insular subnetwork connectivity in patients with SCD was able to correctly classify 83.9% of SCD and CN subjects (χ^2^ = 66.239, *p* < 0.001; Nagelkerke *R*^2^ = 0.777). The ROC curve of each altered insular subnetwork connectivity is presented in Figure 3A. The best-fitting model for this analysis had an area under the ROC curve (AUC) of 0.876, with 81.6% sensitivity and 81.8% specificity.

Next, using binary logistic regression analyses, we found that the best-fitting model combining altered insular subnetwork connectivity in patients with aMCI was able to correctly classify 86.5% of aMCI and CN subjects (χ^2^ = 71.785, *p* < 0.001; Nagelkerke *R*^2^ = 0.635). The ROC curve of each altered insular subnetwork connectivity is displayed in Figure 3B. The best-fitting model had an AUC of 0.887, with 80.4% sensitivity and 83.6% specificity.

### Behavioral Significance of Altered FC Within Insular Subnetworks in Patients With SCD and aMCI

**Figure 4:**
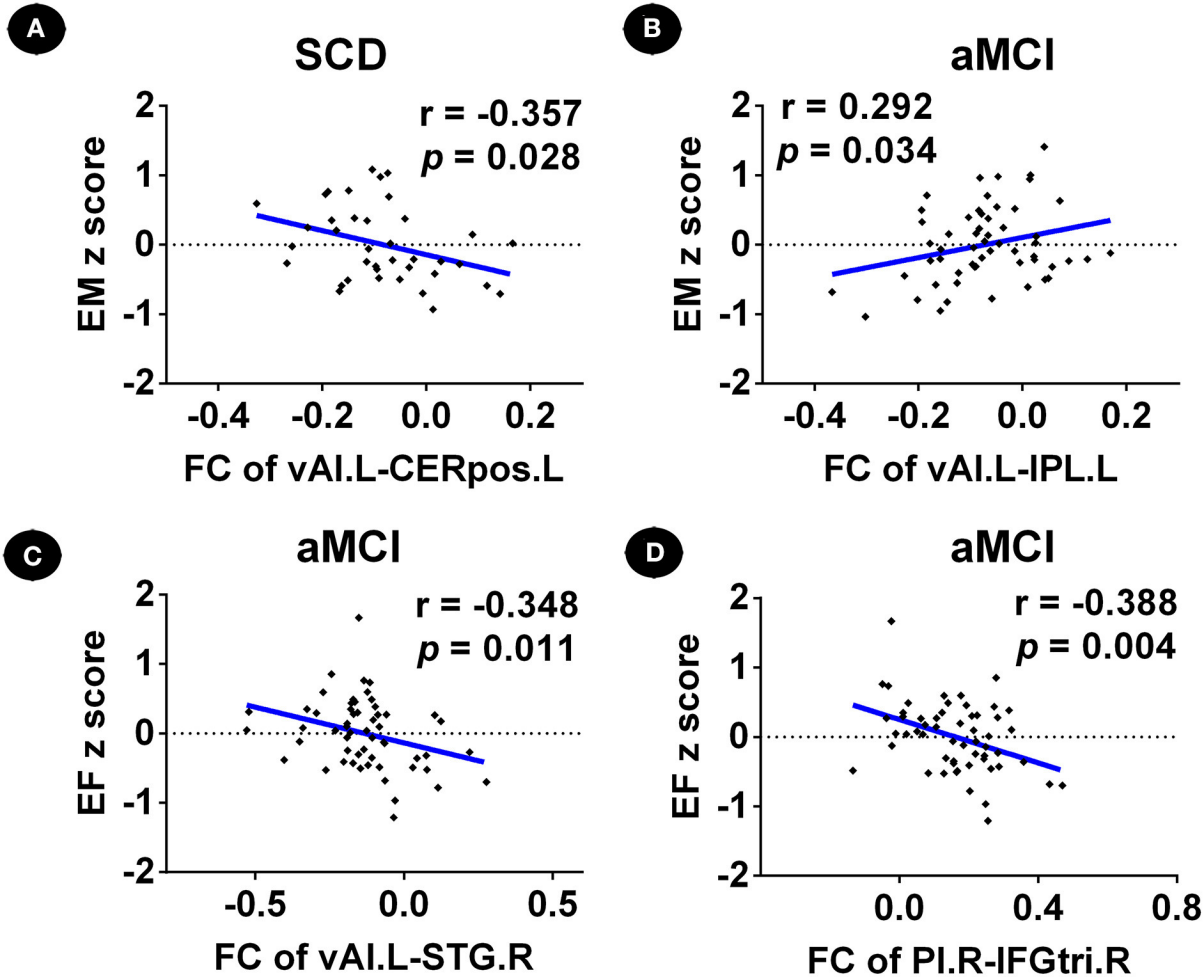
Relationship between abnormal insular subnetwork functional connectivity and cognition in patients with SCD and aMCI. **(A)** Relationship between the insular subnetwork FC and cognition in patients with SCD. **(B–D)** Relationship between the insular subnetwork FC and cognition in patients with aMCI. FC, functional connectivity; CN, normal control; SCD, subjective cognitive decline; aMCI, amnestic mild cognitive impairment; vAI.L, left ventral anterior insula; PI.R, right posterior insula; CERpos.L, left cerebellum posterior lobe; IPL.L, left inferior parietal lobule; STG.R, right superior temporal gyrus; IFGtri.R, right inferior frontal gyrus, triangular part; EM, episodic memory; EF, executive function.

Functional connectivity between the left vAI and left cerebellum posterior lobe in patients with SCD was negatively correlated with episodic memory score (*r* = −0.357, *p* = 0.028) (Figure 4A). Furthermore, FC between the left vAI and left IPL within patients with aMCI was positively correlated with episodic memory score (*r* = 0.292; *p* = 0.034), while FC between left vAI and right STG (*r* = −0.348; *p* = 0.011), and FC between right PI and right IFGtri was negatively correlated with executive function scores (*r* = −0.388, *p* = 0.004) (Figures 4B–D). Furthermore, we did not find any correlation between depression (HAMD scores) and changes in FC in patients with SCD and aMCI (*p* > 0.05).

## Discussion

To the best of our knowledge, this is the first study to evaluate altered FC patterns of insular subnetworks in the pre-clinical spectrum of AD, and investigate the relationship between altered FC and behavioral significance. Binary logistic regression analyses were further applied for the classification of pre-clinical AD stages. According to our study results, we discovered that altered FC, if found, were mainly in overlapping areas among insular subnetworks (vAI, dAI, and PI) (Nomi et al., 2018), part of which is correlated with the abnormal cognition. Nonetheless, similarities and differences do exist between the two pre-clinical AD stages, and this utilization of abnormalities is able to precisely distinguish the spectrum of pre-clinical AD. Taken together, our results could imply that altered FCs within insular subnetworks may act as a biomarker for prompt detection, intervention, and treatment for pre-clinical AD.

### Altered FC Patterns of Insular Subnetworks in Patients With SCD

We detected reduced FC within the L-vAI and L-dAI of cerebellum posterior lobe in patients with SCD compared to CN subjects. Furthermore, early studies discovered that the cerebellum posterior lobe contributes to episodic memory coding (Fliessbach et al., 2007). Additionally, altered FC within this area is closely related to abnormal episodic memory in patients with aMCI (Bai et al., 2011). In accordance with earlier observations, we identified a negative relationship between altered FCs of vAI in the cerebellum posterior lobe and episodic memory. With a gradual deterioration of FC, the episodic memory scores of patients with SCD turn out to increase. One possible explanation for this phenomenon is that while the connection between the cerebellum and insula gradually collapses, the compensatory brain regions (i.e., IFG, MFG, and MTG) become abnormally activated. These excessive intrinsic FCs contribute to transiently improved clinical performance. Notably, a dysfunction in episodic memory is thought to be a typical syndrome of aMCI (Seo and Choo, 2016). Furthermore, apart from aMCI, altered FC in the cerebellum was found to be highly correlated with deteriorated episodic memory performance of patients with SCD. This result implies that SCD may share some similarities in neuronal deterioration with aMCI, and may actually represent as a prodromal aMCI stage.

### Altered FC Patterns of Insular Subnetworks in Patients With aMCI

In comparison to subjects with CN, patients with aMCI presented with reduced FCs of the L-vAI in IPL and in the cerebellum posterior lobe and ACC of L-dAI. IPL is known to be a region of heterogeneity that is associated with diverse brain networks, including DMN, salience network (SAN), and executive control network (ECN) (Wang et al., 2015). IPL has a role in multiple functions, such as the executive and salience effect (Uddin et al., 2011). Moreover, Kwok and Macaluso have demonstrated that IPL may be modulated under the episodic memory retrieval process, which indicates a tight relationship with episodic memory (Kwok and Macaluso, 2015). In addition, Xie et al. (2012) have reported that aberrant insular networks may play a key role in the processes of episodic memory. Consistently, we detected a positive correlation between FCs of L-vAI in IPL and episodic memory. With a reduction in FC, the severity of damage exceeds the degree of compensation, which leads to irreversible disrupted episodic memory performance. This implies decreased neuroactivity between IPL and L-vAI, which eventually causes worsened clinical performance. ACC is engaged in various processes, which includes attention, memory, and emotion (Fillinger et al., 2018). To date, numerous studies have demonstrated strong functional or structural connections between the dorsal ACC and dAI (Chang et al., 2013; Wiech et al., 2014; Ghaziri et al., 2017). Consistently, our result reveals a functional relationship between ACC and dAI, which indicates a potential deterioration mechanism of aMCI.

Convergent altered intrinsic FC remains in MFG, STG, and MTG under L-vAI, and in IFG under R-PI. MFG and MTG both belong to DMN and are relevant in cognitive processes. The abnormally increased FCs of patients with aMCI between these two regions and L-vAI implies possible recruitment of activated DMN for an early functional complement. Additionally, STG is in charge of primary and secondary auditory and lingual processes (Luo et al., 2018). Furthermore, Mwansisya et al. (2017) have reported convergent neuronal abnormalities within activated STG, which is closely related to executive dysfunction in schizophrenia. Consistently, we discovered a negative relationship between executive function and altered FC of L-vAI in STG, which validates an intimate relationship between STG and executive dysfunction of patients with aMCI. These results indicate that aMCI (or AD) may share similarities with regard to psychopathology and compensatory mechanisms.

### Convergent and Divergent Altered FC of Insular Subnetworks in Patients With SCD and aMCI

Subjective cognitive decline and aMCI share both similarities and differences in altered FC of insular subnetworks. To summarize, we found that there were compensatory brain regions in patients with SCD, including MFG, MTG, and IFG. In addition, we also discovered that the compensatory phenomenon exists in STG in patients with aMCI. Furthermore, altered FC in MTG was detected across all three insular subnetworks, suggesting that STG may represent the main compensatory region in patients with SCD. However, compensation in patients with aMCI is more prominent, which further reflects the adaptation of deepened atrophy. With regards to the distribution of affected regions, IFG and MFG belong to the frontal regions while MTG and STG belong to the temporal regions. With the ever-expanding influence of neurotoxicity, researchers have discovered the presence of excessive strengthened intrinsic neuroactivities among the frontal and temporal regions (Qi et al., 2010; Gour et al., 2011; Yang et al., 2018). Combined with our results, we infer that the frontal and temporal regions may serve as the major and the earliest regions that regulate compensatory activities in pre-clinical AD stages. These results suggest a tendency to recruit advanced brain regions to compensate for early functional loss.

In addition, our study showed no insular differences in FC in the SCD group compared to the aMCI group. This is a surprising finding given the clear-cut cognitive differences that are present between the SCD and aMCI groups, as well as the different stages that are attributed to these groups in the clinical and pathological progression of AD. Our results suggest that even though these groups are clinically different, their underlying insular pathology may not be. Previous studies have highlighted the similarities and the overlap of the cerebral amyloid burden between patients with SCD and MCI (Wolfsgruber et al., 2017; Jessen et al., 2018). The explanation may be that SCD, in the presence of AD pathology, may represent the stage at which there is the first subtle cognitive dysfunction in pre-clinical AD (Sperling et al., 2011). At this stage, SCD reflects an individual's experience of this subtle cognitive decline, which is still in a significant compensatory phase (Erk et al., 2011). Furthermore, our previous study discovered that the compensatory phenomenon of neural networks in SCD individuals is associated with clinical cognition measures (Xue et al., 2019). This further indicates that AD pathology occurs in a temporally ordered manner, along with disease progression (Jack et al., 2013; Chen et al., 2019a). Therefore, SCD is considered a highly attractive stage for future early interventions in pre-clinical AD, as the brain function remains largely preserved with intact compensatory processes.

Our results also indicated that SCD and aMCI subjects have significantly higher HAMD scores in comparison to controls, despite the fact none of the subjects were depressed (HAMD score <7). This suggests that depression may be a confounding factor of insular connectivity changes in SCD and aMCI. Recently, Liew (2019) investigated the independent risk factors for neurocognitive disorders associated with depression in a large sample study. Their results suggested that SCD and depression were both independent risks of MCI/dementia, but the combination of depression and SCD was a higher risk compared to SCD alone. However, in order to avoid this confounding factor, all subjects had depression scores of <7. Furthermore, there were no correlations found between depression scores and insular connectivity. Therefore, it is reasonable to speculate that the connectivity changes seen are indicative of SCD and aMCI, and are not representative of a state of depression.

### Distinctly Altered Connectivity Patterns of Insular Subnetworks Within the Separate Spectrum of Pre-clinical AD

Even within the same group, there are differences among vAI, dAI, and PI. Based on our study results, we can infer that vAI is more sensitive with regard to the manifestation of compensation. Furthermore, vAI delivers more evident clinical manifestations of deterioration and compensation compared to dAI and PI and is associated with strong clinical significance. Liu et al. (2018) have reported that vAI, dAI, and PI are closely associated with SAN, ECN, and somatomotor network, respectively, indicating separate engagement in cognitive, affective, and interoceptive processes. The insula is highly associated with neuronal nodes that are related to episodic memory (Sugar and Moser, 2019) and possesses the function of executive control (Menon and Uddin, 2010). Our data have revealed that vAI is recruited in the functioning of episodic memory and executive function. Additionally, our results reveal a strong relationship between executive function and PI. Since there are distinct brain regions located within the hub of insular subnetworks, we infer that FC among these interactive subregions possesses multimodal functions. Under certain circumstances in which a specific function of a subregion is impaired, FC is increased within that region. However, further experimentation is required to verify specific mechanisms. Our preliminary results suggest that altered FC detection in pre-clinical AD patients may reversely play an essential role in revealing the internal relationships within insular subnetworks.

### Precise Classification of the Spectrum of Pre-clinical AD by Model Combining Altered Insular Subnetwork Connectivity

To date, this is the first time that aberrant FC of insular subnetworks has been applied to identify the stages of the spectrum of pre-clinical AD. Utilizing binary logistic regression, we were able to obtain two separate optimal models for each pre-clinical stage, SCD, and aMCI. The accuracy of classification for the SCD model was 83% (81% sensitivity and 81.8% specificity), while the accuracy of the aMCI model was 86.5% (80% sensitivity and 83.6% specificity). Through this comparison, we are able to conclude that the aMCI classification model has higher overall accuracy. While the SCD classification model has increased competence in the identification of real patients, the aMCI classification model possesses a greater capability of excluding healthy individuals. Based on these results, as well as a combination of abnormal FCs in several altered brain regions, which belong to the overlapping areas of vAI, dAI, and PI, we are able to precisely distinguish the spectrum of pre-clinical AD. Therefore, abnormal connectivity among these regions can be utilized as a tool for early clinical diagnosis, and these altered regions can also be used as therapeutic targets for early intervention and treatment.

## Limitation

There are several limitations to our study. Firstly, we have a small sample size, and only 55 CNs, 38 patients with SCD, and 56 patients with aMCI were included in this study. This may have led to a bias of the results. To enhance the statistical power of our results, we applied a non-parametric permutation test, which allows control of the false positive rate in cluster-level inference. Meanwhile, our NBH-ADsnp database is constantly being updated and recruits new volunteers, and therefore, we will further verify our conclusions shortly. Secondly, we studied the behavioral significance of FC abnormalities in its resting state, which has some limitations. The task-based fMRI may be utilized to verify these results and further evaluate the behavioral significance of FC alterations. Finally, we found no differences in FC between SCD and aMCI, which may limit the validity of insular FC measures in staging individuals that are on the spectrum of pre-clinical AD. This may suggest that insular FC features may simply serve as biological markers of disease, rather than markers of disease progression. The reason for these findings may be that aMCI subjects were recruited from the memory clinic and local communities. In the future, we hope to further expand the sample size in order to assess the characteristics of insular FC differences in patients with aMCI from the local community vs. patients enrolled from the memory clinic.

## Conclusion

Subjective cognitive decline and aMCI, part of the spectrum of pre-clinical AD, share some convergent and divergent altered intrinsic connectivity of insular subnetworks, with differences in abnormalities existing among subnetworks. These abnormalities can be applied to accurately identify patients that are within the spectrum of pre-clinical AD, which suggests that aberrant functional connectivity within insular networks may serve as a strong potential biomarker in the diagnosis of pre-clinical AD stages.

## Data Availability Statement

The raw data supporting the conclusions of this article will be made available by the authors, without undue reservation.

## Ethics Statement

The studies involving human participants were reviewed and approved by the responsible Human Participants Ethics Committee of the Affiliated Brain Hospital of Nanjing Medical University. The patients/participants provided their written informed consent to participate in this study.

## Author Contributions

JC, XZ, SW, and HS: study design. SW, HS, GH, CX, WQ, JR, and FZ: data acquisition. SW and HS: data analyses. SW and HS: manuscript preparation. All authors contributed to the article and approved the submitted version.

## Conflict of Interest

The authors declare that the research was conducted in the absence of any commercial or financial relationships that could be construed as a potential conflict of interest.
