# Supplementary material for: Altered Insular Subregional Connectivity Associated With Cognitions for Distinguishing the Spectrum of Pre-clinical Alzheimer's Disease
Source: Front Aging Neurosci. 2021 Feb 10;13:597455. doi: 10.3389/fnagi.2021.597455 (PMC7902797; doi:10.3389/fnagi.2021.597455)
Supplement: Supplementary file 1 [file Data_Sheet_1.pdf]

Altered insular subregional connectivity associated with cognitions for distinguishing the spectrum of preclinical Alzheimer's disease

Siyu Wang[1,2,#], Haiting Sun[3,#], Guanjie Hu[1,2], Chen Xue[4], Wenzhang Qi[4], Jiang Rao[5], Fuquan Zhang[6], Xiangrong Zhang[2,7*], Jiu Chen[1,2*]

[1]Institute of Neuropsychiatry, the Affiliated Brain Hospital of Nanjing Medical University, Fourth Clinical College of Nanjing Medical University, Nanjing, Jiangsu, 210029, China

[2]Institute of Brain Functional Imaging, Nanjing Medical University, Nanjing, 210029, China

[3]Department of Pediatrics, Xijing Hospital, The Fourth Military Medical University, Xi'an, 710032, China

[4]Department of Radiology, the Affiliated Brain Hospital of Nanjing Medical University, Nanjing, 210029, China

[5]Department of rehabilitation, the Affiliated Brain Hospital of Nanjing Medical University, Nanjing, 210029, China

[6]Department of Psychiatry, the Affiliated Brain Hospital of Nanjing Medical University, Nanjing, 210029, China

[7]Department of Geriatric Psychiatry, the Affiliated Brain Hospital of Nanjing Medical University, Nanjing, 210029, China

Running Title: Insular subnetworks across preclinical AD spectrum.

[#] : **Siyu Wang** and **Haiting Sun** have contributed equally to this work (joint first authors).

***Correspondence to**:

Jiu Chen, Institute of Neuropsychiatry, Institute of Brain Functional Imaging, the Affiliated Brain Hospital of Nanjing Medical University, No.264, Guangzhou Road, Gulou District, Nanjing, Jiangsu, 210029, China. E-mail: ericcst@aliyun.com.

Xiangrong Zhang, Department of Geriatric Psychiatry, the Affiliated Brain Hospital of Nanjing Medical University, No.264, Guangzhou Road, Gulou District, Nanjing, Jiangsu, 210029, China. Email: drxrz@hotmail.com.

Supporting Information

SI methods

S.1 NBH-ADsnp database

Data used in this study were obtained from the Nanjing Brain Hospital-Alzheimer's Disease (AD) Spectrum Neuroimaging Project (NBH-ADsnp) database (in-home website: http://192.168.8.100) (Nanjing, China).NBH-ADsnp was derived from an AD Spectrum Neuroimaging Project that was launched in January 2018 by the Institute of Brain Functional Imaging, the Affiliated Brain Hospital of Nanjing Medical University (Nanjing, China). Prof. Jiu Chen, PhD, MD, from the Affiliated Brain Hospital of Nanjing Medical University, served as the principal investigator of NBH-ADsnp. NBH-ADsnp was initiated by Dr. Jiu Chen and Dr. Xiangrong Zhang and was named by Dr. Jiu Chen's research group (discussed by Chen Xue, Guanjie Hu, Wenwen Xu, Wan Liu, Wenzhang Qi, Siyu Wang, Jiani Xu, Shanshan Chen, and finally verified by Jiu Chen and Xiangrong Zhang).NBH-ADsnp is an observational study which includes cross-sectional and longitudinal follow-up components. The goal of NBH-ADsnp is to identify early neuroimaging biomarkers of preclinical Alzheimer's Disease (AD) spectrum {subjective cognitive decline (SCD), amnestic mild cognitive impairment (aMCI),non-amnestic mild cognitive impairment (naMCI), and AD}, to predict disease progression of individuals within the preclinical AD spectrum, and to provide imaging-based targets for individualized intervention to prevent disease deterioration from preclinical stages to the eventually progressed AD. Initially, several hundreds of elderly individuals in NBH-ADsnp, who were all Han Chinese and right-handed, were recruited from hospitals and local communities by advertising and by means of broadcasting. This database used a standardized clinical evaluation protocol that included a medical history interview, neurologic examination, a battery of neurocognitive assessments, and a resting-state MRI scan (T1, T2, 3D T1, DTI, and BOLD) for all participants (normal controls (CN), SCD, naMCI, aMCI, and AD). All subjects and their study partners completed the informed consent process, and study protocols were reviewed and approved by the responsible Human Participants Ethics Committee of the Affiliated Brain Hospital of Nanjing Medical University (No. 2018-KY010-01, No. 2020-KY010-02, No. ChiCTR1900022287, and No. ChiCTR2000034533).

The general eligibility, inclusion, and exclusion criteria for NBH-ADsnp subjects were as follows:

Inclusion criteria of SCD subjects were identified to meet the published SCD research criteria proposed by the Subjective Cognitive Decline Initiative (SCD-I) (Jessen et al., 2014), and the detailed inclusion criteria were described in our previously published study(Xue et al., 2019) as follows: (a) self-reported persistent memory decline, which was confirmed by an informant; (b) Subjective Cognitive Decline Questionnaire (SCD-Q) score > 5 (Cedres et al., 2019; Hao et al., 2017; T. Yan et al., 2018); (c) performance within the normal range on MMSE and MoCA (adjusted for age and education); (d) Clinical Dementia Rating (CDR) = 0; and (e) subjects aged between 50 and 80 years old.

Inclusion criteria of naMCI subjects were described in previous studies (Dunn et al., 2014; Xue et al., 2019) as follows: a) normal overall cognitive function just like aMCI patients; b) the tests scores about memory function are in the normal range while deficits were present in other cognitive domains, including visual spatial function, executive function, language function, and information processing speed; and (c) subjects aged between 50 and 80 years old.

Inclusion criteria of aMCI subjects were identified to meet the diagnostic criteria defined by Peterson et al. (Petersen et al., 1999) as well as to follow the revised consensus standards presented by Winblad et al. (Winblad et al., 2004), and the detailed inclusion criteria were described in our previously published studies (Chen et al., 2019a; Chen et al., 2016b; Xue et al., 2019): (a) memory complaint preferably corroborated by an informant or the subject for more than 3 months; (b) objective memory impairment adjusted for age and educational level; (c) normal general cognitive function of MMSE score equal or above 24; (d) no or minimal impairment in daily living activities; (e) CDR=0.5;(f) subjects aged between 50 and 80 years old; and (g) absence of dementia symptoms that were not sufficient to meet the criteria of the National Institute of Neurological and Communicative Disorders and Stroke or the AD and Related Disorders Association criteria for AD.

Inclusion criteria of CN subjects were as follows: (a) without memory complaints; (b) normal cognitive performance matched for age and education; (c) CDR=0;(d) MMSE ≥ 26; and (e) subjects aged between 50 and 80 years old.(Chen et al., 2019b; Xue et al., 2019).

Detailed exclusion criteria for all subjects were described in our previously published studies (Chen et al., 2019a; Chen et al., 2016b; Xue et al., 2019): (a) a past history of stroke (modified Hachinski Ischemic Scale score > 4), alcoholism, head injury, brain tumors, Parkinson's disease, epilepsy, encephalitis, major depression (excluded by HAMD), or other neurological or psychiatric illnesses (excluded by clinical assessment and case history); (b) major medical illness (e.g., cancer, anemia, thyroid dysfunction, syphilis, or HIV); (c) severe visual or hearing loss; (d) unable to complete neuropsychological tests or with a contraindication for MRI, and (5) T2-weighted MRI showing major changes in white matter (WM), infarction, or other lesions (two experienced radiologists analyzed the scans). None of the patients used any medications.

S.2 Neuropsychological assessments for the NBH-ADsnp database

Neuropsychological assessments were as described in our previously published studies (Chen et al., 2019a; Chen et al., 2016a; Chen et al., 2015; Chen et al., 2019b; Xue et al., 2019). All subjects underwent a standardized clinical interview and comprehensive neuropsychological assessments that were performed by neuropsychologists (Dr. Xue, Qi, and Liu), , including Mini Mental State Examination (MMSE), Montreal Cognitive Assessment (MoCA), Mattis Dementia Rating Scale (MDRS), Auditory Verbal Learning Test - immediate recall (AVLT-IR), Auditory Verbal Learning Test-5-min delayed recall (AVLT-5-min-DR), Auditory Verbal Learning Test-20-min delayed recall (AVLT-20-min-DR), Logical Memory Test-immediate recall (LMT-IR), Logical Memory Test-20-min delayed recall (LMT-20-min-DR), Rey-Osterrieth Complex Figure Test-20-min delayed recall (ROCFT-20min-DR), Clock Drawing Test (CDT), Rey-Osterrieth Complex Figure Test (ROCFT), Verbal Fluency Test (VFT), Digit Span Test (DST), Digital Symbol Substitution Test (DSST), Trail-Making Tests A and B (TMT-A and B), Stroop Color and Word Test A, B, and C, and Semantic Similarity (Similarity) test. These tests were used to evaluate general cognitive function, episodic memory, information processing speed, executive function, and visuo-spatial function, respectively.

S.3 Image acquisition for the NBH-ADsnp database

The NBH-ADsnp data acquisition process was as described in our previously published studies (Chen et al., 2019a; Chen et al., 2016b; Chen et al., 2019c). The details regarding image acquisition parameters in NBH-ADsnp were provided in our

previously published study(Xue et al., 2019).

All MRI data were acquired using a 3.0 Tesla Verio Siemens scanner with an 8-channel head-coil in the Affiliated Brain Hospital of Nanjing Medical University (Nanjing, China). Resting-state functional images were collected when participants were instructed to rest with their eyes open, to not fall asleep, and to not think of anything in particular. The gradient-echo echo-planar imaging (GRE-EPI) sequence included 240 volumes. The parameters were as follows: repetition time (TR) = 2000 ms, echo time (TE) = 30 ms，number of slices = 36, thickness = 4.0 mm, gap = 0 mm, matrix = 64×64, flip angle (FA) = 90°, field of view (FOV) = 220 mm×220 mm, acquisition bandwidth = 100 kHz, voxel size = 3.4×3.4×4 mm^3. The imaging process took approximately 8 minutes.

High-resolution T1-weighted images were acquired by a 3D magnetization-prepared rapid gradient-echo (MPRAGE) sequence. The parameters were as follows: TR = 1900 ms, TE = 2.48 ms, inversion time (TI) = 900 ms, number of slices = 176, thickness = 1.0 mm, gap = 0.5 mm, matrix = 256×256, FA = 9°, FOV = 256 mm×256 mm, voxel size = 1×1×1 mm^3. The imaging process took approximately 4.26 minutes.

Additionally, routine axial T2-weighted images were acquired to rule out subjects with major changes in WM, cerebral infarction or other lesions using flair sequence as below: TR = 8400 ms, TE = 94 ms, FA= 150°, acquisition matrix = 256×256, FOV = 230×230 mm, thickness = 5.0 mm, gap = 0 mm, and number of slices = 20. The imaging process took approximately 2.50 minutes.

S.4 fMRI image preprocessing

All fMRI data were preprocessed using MATLAB2015b (http://www.mathworks.com/products/matlab/) and DPABI image processing software (C. G. Yan et al., 2016). The image processing procedure was as previously described (C. G. Yan et al., 2013)as follows: the first ten volumes were discarded to reduce the instability of MRI signal. Corrections were performed for the intra-volume acquisition time differences among slices and inter-volume motion effects during the scan (slice timing correction and head motion correction) (Power et al., 2012; Van Dijk et al., 2012). Participants with excessive head motion (cumulative translation or rotation > 3.0 mm or 3.0°) were excluded. Individual functional and structural images were co-registered. The Diffeomorphic Anatomical Registration Through Exponentiated Lie Algebra (DARTEL) algorithm was used to normalize and segment the structural images into GM, WM and cerebrospinal fluid (CSF) partitions(Ashburner et al., 2009). We next used a Friston 24-parameter model (i.e., 6 head motion parameters, 6 head motion parameters one time point before, and the 12 corresponding squared items) to regress out head motion effects from the realigned data (Friston et al., 1996). The WM, CSF, and the global signals as well as the linear trend were also regressed as nuisance covariates (Brady et al., 2019). After realigning, slice timing correction, and co-registration, framewise displacement (FD) was calculated for all resting state volumes (Power et al., 2012). All volumes with a FD greater than 0.2 mm were regressed out as nuisance covariates (Brady et al., 2019). Any scan with 50% volumes removed was discarded (Brady et al., 2019). After nuisance covariate regression, functional images were normalized by DARTEL into MNI space (resampling voxel size, 3 × 3 × 3 mm^3) and then spatially smoothed by a Gaussian kernel of 6 mm^3 full-width at half maximum (FWHM) to reduce spatial noise. Temporal band-pass filtering (0.01–0.1 Hz) was applied to reduce the effect of low-frequency drifts and high-frequency physiological noise. Voxels within a group GM mask created by DARTEL were used for further analyses.

SI results

S.1 ANCOVA results from insular subnetwork functional connectivity among SCD patients, aMCI patients, and CN

Fig. S1

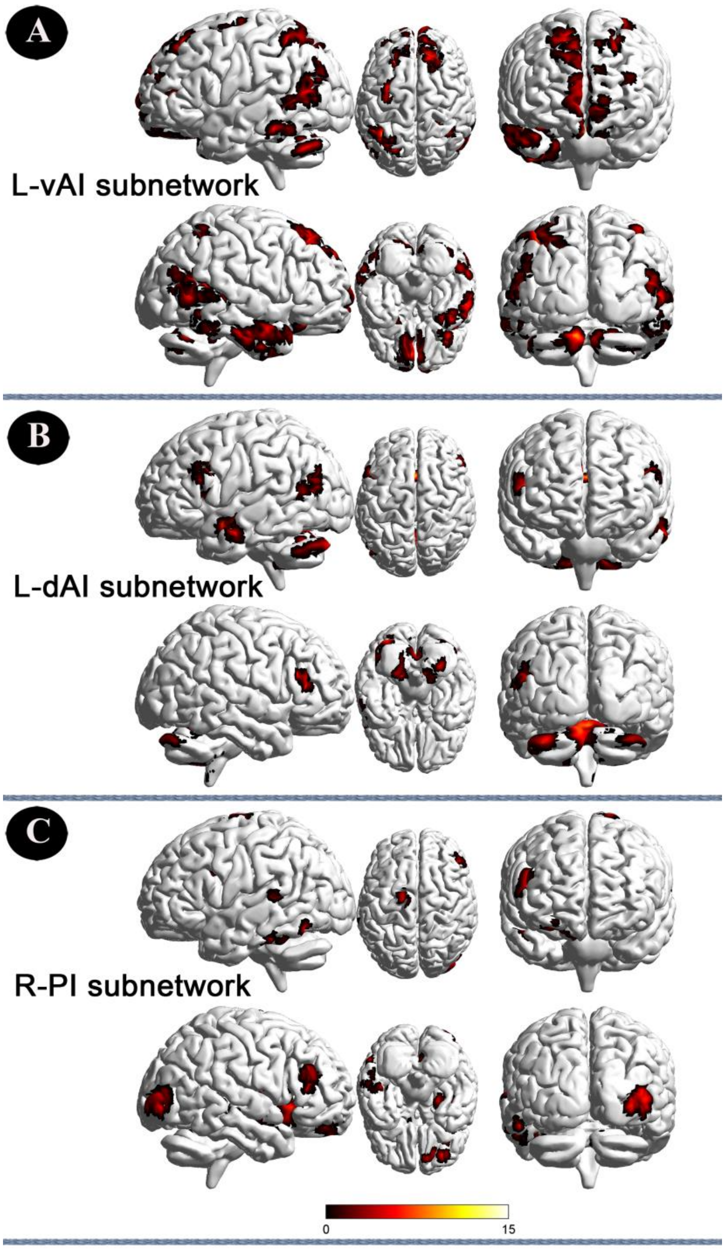

Fig. S1. ANCOVA results from insular subnetwork functional connectivity among SCD patients, aMCI patients, and CN. (A, B, C) indicating brain different regions of the functional connectivity of left vAI, left dAI, right PI subregions among CN, SCD, and aMCI. Note: only the subnetworks showing differences among groups are shown here. All results were displayed after controlling age, sex, education, ITV, and FD at a threshold of $p < 0.001$ using TFCE correction with cluster size > 270 mm^3.

Abbreviations: CN, normal control; SCD, subjective cognitive decline; aMCI, amnestic mild cognitive impairment; L-vAI, left ventral anterior insula; L-dAI, left dorsal anterior insula; R-PI, right posterior insula; TFCE, threshold-free cluster enhancement; ITV, intracranial volume; FD, framewise displacement; ANCOVA, analysis of covariance.

Supplementary tables

Table S1.

Seeds and MNI coordinates

Seeds	MNI coordinates		
	x	y	z
L-vAI	− 33	13	-7
R-vAI	32	10	-6
L-dAI	-38	6	2
R-dAI	35	-11	6
L-PI	-38	-6	5
R-PI	35	-11	6

Note: our definition of insular subregions referred to recent studies (Deen et al., 2011; Lu et al., 2020; Peng et al., 2018).

Abbreviations: L-vAI, left and right ventral anterior insula; R-vAI, right ventral anterior insula; L-dAI, left dorsal anterior insula; R-dAI, right dorsal anterior insula; L-PI, left posterior insula; R-PI, right posterior insula; MNI, montreal neurological institute. Coordinates for seeds in the right and left hemispheres are defined in the MNI stereotaxic space.

Table S2.

Detailed raw scores and corresponding Z scores of individual neuropsychological tests for all subjects

Items		CN	SCD	aMCI		
		n=55	n=38	n=56	*F* values	*p* values
**MDRS**						
Attention	raw score	36.75(0.52)	36.53(0.73)	35.84(1.96)	7.253	0.001*
	Z score	0.29(0.38)	0.13(0.54)	-0.38(1.45)	7.253	0.001*
Initiation/Preservation	raw score	36.60(1.24)	36.66(1.58)	35.52(2.96)	4.752	0.010*
	Z score	0.18(0.57)	0.21(0.73)	-0.32(1.36)	4.752	0.010*
Conceptual	raw score	38.42(1.01)	38.37(1.05)	37.34(2.70)	5.764	0.004*
	Z score	0.22(0.53)	0.19(0.55)	-0.35(1.42)	5.764	0.004*
Construct	raw score	5.98(0.13)	5.87(0.41)	5.82(0.61)	1.971	0.143
	Z score	0.20(0.31)	-0.06(0.95)	-0.16(1.39)	1.971	0.143
Memory	raw score	23.71(1.29)	22.92(1.75)	21.91(2.13)	14.537	0.000*
	Z score	0.46(0.67)	0.05(0.91)	-0.48(1.11)	14.537	0.000*
Total	raw score	141.46(2.33)	140.37(3.05)	136.45(6.67)	37.224	0.000*
	Z score	0.42(0.46)	0.21(0.60)	-0.56(1.31)	37.224	0.000*
**Episodic memory**						
AVLT-IM	raw score	19.15(4.36)	18.66(4.22)	15.13(4.29)	13.946	0.000*
	Z score	0.35(0.94)	0.25(0.91)	-0.51(0.92)	13.946	0.000*
AVLT-5-min-DR	raw score	6.35(2.20)	6.26(1.90)	4.23(2.29)	16.203	0.000*
	Z score	0.34(0.93)	0.31(0.80)	-0.55(0.96)	16.203	0.000*
AVLT-20-min-DR	raw score	6.30(1.94)	6.32(2.12)	4.23(2.29)	28.106	0.000*
	Z score	0.40(0.73)	0.41(0.80)	-0.68(0.99)	28.106	0.000*
LMT-IR	raw score	6.29(3.25)	5.68(3.31)	4.14(2.80)	6.955	0.001*
	Z score	0.30(1.00)	0.11(1.02)	0.37(0.87)	6.955	0.001*
LMT-20-min-DR	raw score	4.95(3.07)	4.82(2.83)	3.01(2.39)	8.085	0.000*
	Z score	0.26(1.06)	0.22(0.98)	-0.40(0.83)	8.085	0.000*
ROCFT-20-min-DR	raw score	16.51(5.49)	18.04(6.15)	12.88(7.03)	8.697	0.000*
	Z score	0.15(0.83)	0.38(0.93)	-0.40(0.83)	8.697	0.000*
**Visuospatial function**						
ROCFT	raw score	34.78(2.11)	35.00(1.43)	33.21(3.46)	7.264	0.001*
	Z score	0.20(0.78)	0.28(0.53)	-0.38(1.29)	7.264	0.001*
CDT	raw score	9.36(1.21)	9.47(0.95)	8.84(1.20)	4.417	0.014*
	Z score	0.14(1.03)	0.24(0.81)	-0.30(1.03)	4.417	0.014*
**Information processing speed**						
DSST	raw score	44.16(10.23)	42.42(11.40)	33.65(9.56)	16.151	0.000*
	Z score	0.39(0.90)	0.23(1.01)	-0.54(0.85)	16.151	0.000*
TMT-A	raw score	51.62(12.80)	53.68(13.98)	69.68(40.63)	7.141	0.001*
	Z score	0.31(1.09)	0.15(0.84)	-0.41(0.87)	7.141	0.001*
Stoop-A	raw score	25.06(4.69)	25.74(5.12)	27.66(5.30)	3.943	0.021*
	Z score	0.22(0.91)	0.12(1.12)	-0.30(0.95)	3.943	0.021*
Stoop-B	raw score	41.69(9.85)	41.40(11.22)	47.41(13.83)	4.269	0.016*
	Z score	0.15(0.93)	0.21(0.96)	-0.29(0.95)	4.269	0.016*
**Executive function**						
VFT	raw score	26.27(6.71)	26.26(6.59)	22.64(7.71)	4.593	0.012*
	Z score	0.19(0.93)	0.19(0.91)	-0.31(1.06)	4.593	0.012*
DST	raw score	12.86(1.70)	13.00(1.71)	11.38(1.79)	13.752	0.000*
	Z score	0.28(0.90)	0.35(0.91)	-0.51(0.95)	13.752	0.000*
TMT-B	raw score	122.56(31.45)	135.32 (42.43)	177.25(64.34)	13.946	0.000*
	Z score	0.39(0.86)	0.18(1.05)	-0.51(0.88)	13.946	0.000*
Stoop-C	raw score	77.62(20.25)	74.68(21.64)	91.77(29.91)	6.938	0.001*
	Z score	0.14(0.94)	0.33(1.05)	-0.35(0.93)	6.938	0.001*

Similarity	raw score	19.06(3.52)	19.50(3.21)	14.63(4.85)	23.254	0.000*
	Z score	0.34(0.77)	0.44(0.70)	-0.63(1.06)	23.254	0.000*

Data are presented as the mean (standard deviation, SD). Abbreviations: MDRS, Mattis Dementia Rating Scale; AVLT-IR, Auditory Verbal Learning Test-immediate recall; AVLT-5-min-DR, Auditory Verbal Learning Test-5-minute delayed recall; AVLT-20-min-DR, Auditory Verbal Learning Test-20-minute delayed recall; LMT-IR, Logical Memory Test-immediate recall; LMT-20-min-DR, Logical Memory Test-20-minute delayed recall; ROCFT-20min-DR, Rey-Osterrieth Complex Figure Test-20-minute delayed recall; CDT, Clock Drawing Test; ROCFT, Rey-Osterrieth Complex Figure Test; DSST, Digital Symbol Substitution Test; TMT-A, Trail Making Test-A; Stroop, Stroop Color and Word Test; VFT, Verbal Fluency Test; DST, Digit Span Test; TMT-B, Trail Making Test-B; Similarity, Semantic Similarity Test. * Significant differences are found among three groups. The p values are obtained by ANOVA. Notably, to improve the statistical power, this study used a re-sampling method of stationary bootstrap (10,000 bootstrap samplings) to obtain significance.

References

Ashburner, J., & Friston, K. J. (2009). Computing average shaped tissue probability templates. *Neuroimage, 45*(2), 333-341. doi:10.1016/j.neuroimage.2008.12.008

Brady, R. O., Jr., Gonsalvez, I., Lee, I., Ongur, D., Seidman, L. J., Schmahmann, J. D., et al. (2019). Cerebellar-Prefrontal Network Connectivity and Negative Symptoms in Schizophrenia. *Am J Psychiatry*, appiajp201818040429. doi:10.1176/appi.ajp.2018.18040429

Cedres, N., Machado, A., Molina, Y., Diaz-Galvan, P., Hernandez-Cabrera, J. A., Barroso, J., et al. (2019). Subjective Cognitive Decline Below and Above the Age of 60: A Multivariate Study on Neuroimaging, Cognitive, Clinical, and Demographic Measures. *J Alzheimers Dis, 68*(1), 295-309. doi:10.3233/JAD-180720

Chen, J., Chen, G., Shu, H., Chen, G., Ward, B. D., Wang, Z., et al. (2019a). Predicting progression from mild cognitive impairment to Alzheimer's disease on an individual subject basis by applying the CARE index across different independent cohorts. *Aging (Albany NY), 11*(8), 2185-2201. doi:10.18632/aging.101883

Chen, J., Duan, X., Shu, H., Wang, Z., Long, Z., Liu, D., et al. (2016a). Differential contributions of subregions of medial temporal lobe to memory system in amnestic mild cognitive impairment: insights from fMRI study. *Sci Rep, 6*, 26148. doi:10.1038/srep26148

Chen, J., Shu, H., Wang, Z., Liu, D., Shi, Y., Zhang, X., et al. (2015). The interaction of APOE genotype by age in amnestic mild cognitive impairment: a voxel-based morphometric study. *J Alzheimers Dis, 43*(2), 657-668. doi:10.3233/JAD-141677

Chen, J., Shu, H., Wang, Z., Zhan, Y., Liu, D., Liao, W., et al. (2016b). Convergent and divergent intranetwork and internetwork connectivity patterns in patients with remitted late-life depression and amnestic mild cognitive impairment. *Cortex, 83*, 194-211. doi:10.1016/j.cortex.2016.08.001

Chen, J., Shu, H., Wang, Z., Zhan, Y., Liu, D., Liu, Y., et al. (2019b). Intrinsic connectivity identifies the sensory-motor network as a main cross-network between remitted late-life depression- and amnestic mild cognitive impairment-targeted networks. *Brain Imaging Behav*. doi:10.1007/s11682-019-00098-4

Chen, J., Yan, Y., Gu, L., Gao, L., & Zhang, Z. (2019c). Electrophysiological Processes on Motor Imagery Mediate the Association Between Increased Gray Matter Volume and Cognition in Amnestic Mild Cognitive Impairment. *Brain Topogr*. doi:10.1007/s10548-019-00742-8

Deen, B., Pitskel, N. B., & Pelphrey, K. A. (2011). Three systems of insular functional connectivity identified with cluster analysis. *Cereb Cortex, 21*(7), 1498-1506. doi:10.1093/cercor/bhq186

Dunn, C. J., Duffy, S. L., Hickie, I. B., Lagopoulos, J., Lewis, S. J., Naismith, S. L., et al. (2014). Deficits in episodic memory retrieval reveal impaired default mode network connectivity in amnestic mild cognitive impairment. *Neuroimage Clin, 4*, 473-480. doi:10.1016/j.nicl.2014.02.010

Friston, K. J., Williams, S., Howard, R., Frackowiak, R. S., & Turner, R. (1996). Movement-related effects in fMRI time-series. *Magn Reson Med, 35*(3), 346-355.

Hao, L., Wang, X., Zhang, L., Xing, Y., Guo, Q., Hu, X., et al. (2017). Prevalence, Risk Factors, and Complaints Screening Tool Exploration of Subjective Cognitive Decline in a Large Cohort of the Chinese Population. *J Alzheimers Dis, 60*(2), 371-388. doi:10.3233/JAD-170347

Jessen, F., Amariglio, R. E., van Boxtel, M., Breteler, M., Ceccaldi, M., Chetelat, G., et al. (2014). A conceptual framework for research on subjective cognitive decline in preclinical Alzheimer's disease. *Alzheimers Dement, 10*(6), 844-852. doi:10.1016/j.jalz.2014.01.001

Lu, L., Li, F., Chen, H., Wang, P., Zhang, H., Chen, Y. C., et al. (2020). Functional connectivity dysfunction of insular subdivisions in cognitive impairment after acute mild traumatic brain injury. *Brain Imaging Behav*. doi:10.1007/s11682-020-00288-5

Peng, X., Lin, P., Wu, X., Gong, R., Yang, R., & Wang, J. (2018). Insular subdivisions functional connectivity dysfunction within major depressive disorder. *J Affect Disord, 227*, 280-288. doi:10.1016/j.jad.2017.11.018

Petersen, R. C., Smith, G. E., Waring, S. C., Ivnik, R. J., Tangalos, E. G., & Kokmen, E. (1999). Mild cognitive impairment: clinical characterization and outcome. *Arch Neurol, 56*(3), 303-308.

Power, J. D., Barnes, K. A., Snyder, A. Z., Schlaggar, B. L., & Petersen, S. E. (2012). Spurious but systematic correlations in functional connectivity MRI networks arise from subject motion. *Neuroimage, 59*(3), 2142-2154. doi:10.1016/j.neuroimage.2011.10.018

Van Dijk, K. R., Sabuncu, M. R., & Buckner, R. L. (2012). The influence of head motion on intrinsic functional connectivity MRI. *Neuroimage, 59*(1), 431-438. doi:10.1016/j.neuroimage.2011.07.044

Winblad, B., Palmer, K., Kivipelto, M., Jelic, V., Fratiglioni, L., Wahlund, L. O., et al. (2004). Mild cognitive impairment--beyond controversies, towards a consensus: report of the International Working Group on Mild Cognitive Impairment. *J Intern Med, 256*(3), 240-246. doi:10.1111/j.1365-2796.2004.01380.x

Xue, C., Yuan, B., Yue, Y., Xu, J., Wang, S., Wu, M., et al. (2019). Distinct Disruptive Patterns of Default Mode Subnetwork Connectivity Across the Spectrum of Preclinical Alzheimer's Disease. *Front Aging Neurosci, 11*, 307. doi:10.3389/fnagi.2019.00307

Yan, C. G., Craddock, R. C., Zuo, X. N., Zang, Y. F., & Milham, M. P. (2013). Standardizing the intrinsic brain: towards robust measurement of inter-individual variation in 1000 functional connectomes. *Neuroimage, 80*, 246-262. doi:10.1016/j.neuroimage.2013.04.081

Yan, C. G., Wang, X. D., Zuo, X. N., & Zang, Y. F. (2016). DPABI: Data Processing & Analysis for (Resting-State) Brain Imaging. *Neuroinformatics, 14*(3), 339-351. doi:10.1007/s12021-016-9299-4

Yan, T., Wang, W., Yang, L., Chen, K., Chen, R., & Han, Y. (2018). Rich club disturbances of the human connectome from subjective cognitive decline to Alzheimer's disease. *Theranostics, 8*(12), 3237-3255. doi:10.7150/thno.23772
